# Differences in Gender and Overall Survival for Temperature-Sensitive TP53 Mutations in Gastroesophageal Cancer

**DOI:** 10.3390/medicina60111901

**Published:** 2024-11-20

**Authors:** Danial H. Shaikh, Margaret Park, Jiandong Chen, Jeffrey Huang, Mark S. Friedman, Aamir N. Dam, Anjuli K. Luthra, Saraswathi Cappelle, Luis R. Pena, Jennifer B. Permuth, Shaffer R. S. Mok

**Affiliations:** 1 Department of GI Oncology, Moffitt Cancer Center, Tampa, FL 33612, USA; 2 Department of Bioinformatics and Biostatistics, Moffitt Cancer Center, Tampa, FL 33612, USA; 3 Molecular Oncology Department, Moffitt Cancer Center, Tampa, FL 33612, USA; 4 Department of Anesthesiology & HOB, Moffitt Cancer Center, Tampa, FL 33612, USA

**Keywords:** TP53 mutations, temperature-sensitive (TS), gastroesophageal cancer, overall survival, gender differences

## Abstract

*Background and Objectives*: Temperature-sensitive (TS) mutants of TP53 are thermally unstable, unfolded, and inactive at body temperature but can be refolded and reactivated at sub-physiological temperatures. TS TP53 may be amenable for functional rescue by hypothermia or structure-stabilizing drugs, and may retain low-level transcriptional activity at 37 °C. TP53 mutations are observed in 47% of all esophageal cancers (ECs) and 25% to 40% of gastric cancers (GCs). We aimed to investigate the trends and outcomes of EC and GC with TS TP53 mutations using cBioportal. We hypothesize that TS TP53 mutants in EC and GC present a unique prognostic profile distinct from non-TS TP53 mutants, potentially affecting overall survival and cancer progression. *Materials and Methods*: We identified 1924 patients from cBioportal with GC or EC, harboring any TP53 mutation. Patients were then stratified based on the TP53 temperature sensitivity according to a recently reported functional analysis of its activity. Patients were also stratified based on a history of Barrett’s esophagus (BE), cancer stage, sex, and race. We then compared populations (TS vs. non-TS TP53) to assess differences and evaluated survival outcomes. *Results*: Males represented 77% of the cohort, and 51.6% of the samples were from patients with stage IV cancer. No association was found between TS vs. non-TS mutational status and BE, cancer stage, or race. Interestingly, a significantly higher proportion of females (22.9%) than males (14.5%) displayed a TS TP53 mutation (*p* = 0.012). No significant difference was seen in overall survival between the TS and non-TS mutations capable of ≥50% growth suppression at 32 °C (median = 33 vs. 28 months, *p* = 0.36). This trend was also observed when the patients were filtered based on cancer location. The median survival for EC was 32.5 months compared to 33 months (*p* = 0.67). In cases of GC, median survival times could not be determined due to the insufficient number of events. *Conclusions*: Although no statistical significance was observed, a decrease in overall survival for patients with TS TP53 mutations was noted. The result is counterintuitive given that TS mutants have less severe structural destabilization and suggests TS TP53 mutations may have a unique prognostic value that warrants further investigation.

## 1. Introduction

The TP53 gene on chromosome 17p is a tumor suppressor gene that plays a crucial role in maintaining genomic stability. It acts as a “guardian of the genome” by inducing cell cycle arrest and DNA repair in response to oncogenic stressors, thus preventing cancer development. Mutations in the TP53 gene are common in many types of cancer. They can accumulate non-functional or oncogenic p53 protein (i.e., gain-of-function), contributing to tumor development and progression [1].

Esophageal adenocarcinoma (EAC) and gastric cancer (GC) are two types of cancer that frequently exhibit TP53 mutations. In EAC, TP53 mutations are observed in almost half of all tumors, while in GC, both intestinal and diffuse types, TP53 mutations can range between 25% and 40%, respectively [2]. Mutations affect histologic differentiation, invasion, and regional lymph node metastasis. Past research indicates that TP53 mutations within GC correlates with reduced overall and relapse-free survival rates and are also associated with recurrence [3]. The role of TP53 as a prognostic marker of esophageal squamous cell carcinoma is controversial [4].

TP53 gene polymorphisms have been linked to cancer development and prognosis; in particular, the codon 72 arginine allele (72 arg/arg) has a strong association to increased gastric cancer susceptibility [5,6]. The rate of p53 overexpression (a surrogate for point mutations) in early GC, as detected by immunohistochemistry, ranges from 13 to 54% [7]. In addition, TP53 gene mutations are found in a significant percentage of advanced GC, ranging from 50% to 77% [8]. Although the clinical relevance of TP53 mutations in GC is still unclear, recent studies have suggested that the position of the TP53 mutation may have an impact on clinical outcomes. Specifically, patients with mutations in the turn regions of TP53 tend to have poorer prognoses than those with mutations in other regions [9]. TP53 mutations also play a significant role in the development and progression of Barrett’s esophagus and EAC [10]. These mutations are early events in the molecular pathogenesis of adenocarcinomas in Barrett’s, and are seen in approximately 50% of these patients [11]. They are associated with a progressive increase in cell proliferation and p53 expression during the malignant transformation of the esophageal mucosa [12].

More than 800 different TP53 point mutations have been detected in cancer. However, most studies of mutant TP53 have focused on the gain-of-function oncogenic activities of a few hotspot mutants (codon R175, G245, R248, R273, R282). Despite their high frequency of appearance in cancer, hotspot mutations collectively only account for ~30% of mutant TP53 cases. The remaining 70% of mutant TP53 cases comprise many other point mutations, each detected at a much lower frequency, thus attracting less attention individually. A unique but under-studied subset of TP53 mutations is the temperature-sensitive (TS) mutants. TS TP53 mutations are thermally unstable, unfolded, and inactive at body temperature but can be refolded and reactivated at sub-physiological temperatures [13]. Recent studies have shown that TS TP53 is uniquely amenable to functional rescue by hypothermia or structure-stabilizing drugs [13,14,15]. Unlike non-TS mutants, TS TP53 mutants suffer relatively mild disruptive mutations in the hydrophobic core of the DNA binding domain (DBD) [16]. The minor deficiency in thermostability in the DBD can be compensated by lowering the temperature within a physiologically tolerable range (35–32 °C) and regaining wild-type-like conformation and DNA binding activity [14]. This feature makes specific TS mutants rescuable using cysteine crosslinking agents such as arsenic trioxide, increasing the DBD thermostability [17]. Furthermore, all TS mutants can be efficiently activated at a clinically achievable hypothermia temperature of 32 °C.

Although all tumors select for significant loss of TP53 activity through mutations in the DBD, a recent study showed many TS TP53 mutants retain a measurable level of basal activity at 37 °C, as expected from their less disruptive mutations (Chen lab, manuscript submitted). Furthermore, specific mild TS mutants regain substantial transcriptional activity at 35 °C, suggesting tumors with TS mutations may experience residual p53 tumor suppressor activity with normal body temperature fluctuations. Therefore, we are interested in determining whether TS TP53 mutations are associated with different clinical outcomes when examined separately from non-TS modifications. In this report, we investigated the trends and outcomes of EC and GC (adenocarcinoma histologies only) with TS TP53 mutations using open-source cancer genomic data—cBioportal (New York, NY, USA). This analysis aims to better understand the molecular mechanisms underlying these tumors and identify unique patient characteristics amenable to p53-targeted therapies in the future.

## 2. Materials and Methods

### 2.1. Data Acquisition

Clinical data and TP53 mutation status were downloaded for samples in both the esophageal and stomach cancer cohorts from cBioportal. Inclusion criteria include filtered samples to only those with detectable TP53 mutations and adenocarcinoma histology. For outcomes and statistical analyses, we divide the cohort into 3 groups: (1) adenocarcinoma histology (gastric and esophageal, “Adeno” group) comprising Esophageal Adenocarcinoma, Diffuse Type Stomach Adenocarcinoma, Esophagogastric Adenocarcinoma, Intestinal Type Stomach Adenocarcinoma, Mucinous Stomach Adenocarcinoma, Papillary Stomach Adenocarcinoma, Signet Ring Cell Carcinoma of the Stomach, Stomach Adenocarcinoma, Tubular Stomach Adenocarcinoma; (2) esophageal adenocarcinoma (“EC” group) comprising Esophageal Adenocarcinoma histology; and (3) gastric adenocarcinoma (“GC” group) comprising Diffuse Type Stomach Adenocarcinoma, Esophagogastric Adenocarcinoma, Intestinal Type Stomach Adenocarcinoma, Mucinous Stomach Adenocarcinoma, Papillary Stomach Adenocarcinoma, Signet Ring Cell Carcinoma of the Stomach, Stomach Adenocarcinoma, Tubular Stomach Adenocarcinoma histologies.

### 2.2. Survival Analysis

Patients were classified based on their TP53 temperature sensitivity, which was determined through functional analysis as reported by Tang et al. [13]. They constructed a mutant library using the U937 cancer cell line (TP53−/−) harboring 815 p53-missense mutations in cancer (covering 95.85% of p53-missense mutation cases in the TP53 database maintained by the International Agency for Research on Cancer). The cell library was cultured at either 32 °C or 37 °C for seven days, followed by determining the abundance of p53 variants using next-generation sequencing. Activity of TP53 was calculated as sequencing reads at 32 °C and at 37 °C as a measure of growth inhibition at 32 °C. Patients were stratified into two groups based on their mutant TP53 activity at 32 °C using the following cutoffs: <50% versus ≥50% (low stringency, i.e., mutants capable of reducing cell viability by >50% at 32 °C) and <33% versus ≥33% (high stringency, i.e., mutants capable of reducing cell viability by >77% at 32 °C). Statistical tests were then carried out on the all adenocarcinoma group based on both cutoffs. However, due to the extremely small sample sizes in the high-stringency temperature-sensitive group, statistical analyses were not conducted for esophageal and gastric cancers separately. TP53 mutations not found in this manuscript [13] were excluded.

### 2.3. Statistical Analysis

First, we attempted to assess differences in proportions for dichotomized stringency groups described above (low stringency and high stringency). Contingency tables were generated based on TP53 temperature-sensitive vs. temperature-insensitive status and Barrett’s esophagus status (for the EC group), sex, stage or race. A Cochrane–Armitage trend test was used for ordinal variables (stage) and a Chi-square test for all other variables (sex, BE status, or race). In a follow-up study, Kaplan–Meier survival analysis was performed using the ‘survival’ package and plotted using the ‘survminer’ package for R Project for Statistical Computing. Other statistical analyses were performed using ‘DescTools’ (Cochrane–Armitage) or base R (chi square), version 4.2.1 (The R Foundation for Statistical Computing, Vienna, Austria).

## 3. Results

### 3.1. Characteristics of the Cohort

We identified 1924 patients from cBioportal with GC or EC harboring any TP53 mutation and filtered to adenocarcinoma histologies. In overall agreement with other similar cancer cohorts [18], there was a distinct overrepresentation of males (77.2%) and roughly half were of esophageal origin (52.8%). In alignment with other cohorts, white/Caucasian race represented the majority (87%) and roughly 75% of patients were late-stage (stage III/IV). We observed a mean age at diagnosis of 63 years. Another salient finding is that, while ~17% of all TP53 mutations have been reported to be temperature-sensitive, we observe a frequency of 0.38 (38%) in our cohort, although this may be due to the fact that we filtered out all non-categorizable TP53 mutations based on Tang et al.’s analysis [13]. See Table 1 for a summary of cohort characteristics.

### 3.2. Survival

**Figure 1:**
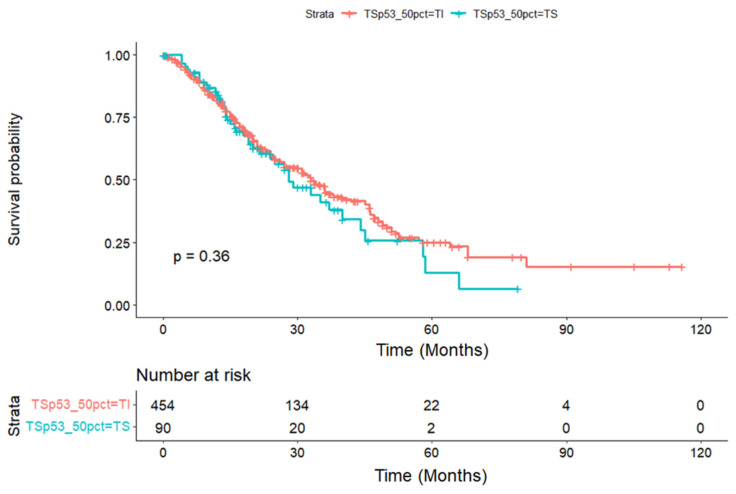
Kaplan–Meier curve demonstrating the overall survival analysis of the temperature sensitive vs. insensitive TP53 mutation at an activity of ≥50% for all adenocarcinoma histologies (‘Adeno’ group). Time is calculated in months from diagnosis. *p*-values were calculated using the Kaplan–Meier estimation.

No significant differences were detected for overall survival between TS and temperature-insensitive mutants for the 50th percentile cutoff (refer to Figure 1 for the Kaplan–Meier curve for the ‘Adeno’ group) in any of the experimental groups (‘Adeno’, ‘EC’, or ‘GC’). For the 50% cutoff analysis not stratified by primary site, a survival median of 33 months (CI: 28–38) was observed for TI and 28 months (CI: 24–44) for the TS mutant patients (*p* = 0.36). For the ‘EC’ group, a median of 32.5 months (CI: 27–38) for TI and 33 months (CI: 25–58) for TS mutants was observed (*p* = 0.67). For a gastric primary site, medians could not be determined due to a low number of events.

Intriguingly, although there were very low numbers of females, when patients were stratified by both gender and temperature sensitivity status, we found that females with temperature-sensitive TP53 mutations demonstrated significantly reduced median survival times (TS = 21.4 months [CI: 13.6-NA] versus TI = 48.0 months [CI: 24.3-NA], BH-adjusted pairwise comparison *p*-value = 0.03) as compared to males (33 months for both TS and TI mutational status, BH-adjusted pairwise comparison *p*-value = 0.887).

Survival analysis was not performed for individual primary sites at the 33% cutoff due to fewer than 2 events occurring for the temperature-sensitive group. See Supplemental Table S1 for survival medians (months) with lower and upper confidence intervals.

### 3.3. Subgroup Analyses

No significant differences in proportion were found via either trend or chi-square test between TP53 TS and TI mutational status and BE status, cancer stage, or race (see Supplemental Table S2). Interestingly, in both the adenocarcinoma group (not subset by primary site) and the esophageal adenocarcinoma (EC) group, a significantly higher proportion of females (22.5% and 28.2%, respectively) displayed a temperature-sensitive TP53 mutation with activity greater than 50% at both 32 °C and 37 °C, compared to males (14.5% and 14.3%, respectively). No differences were observed when the high stringency cutoff (33%) was used.

## 4. Discussion

Our findings highlight a notable gender-specific difference in temperature-sensitive TP53 mutations in gastroesophageal cancer, which may have prognostic implications for patient management. This suggests that future treatment strategies might need to consider gender-specific approaches. We chose to focus on TS TP53 mutants because of their unique characteristics, such as a milder deficit in thermostability and the ability to retain residual transcriptional activity at body temperature. These traits make them particularly intriguing for understanding their distinct role and potential impact on cancer prognosis. Previous research has shown that approximately 11% to 15% of tumor-derived p53 mutants are temperature-sensitive, functioning similarly to wild-type p53 at 32 °C [14]. Shiraishi et al. screened a p53 library of 2314 missense mutations using a yeast-based p53 functional assay and identified 142 TS TP53 mutants, 131 of which were previously unreported [19]. They found that these TS TP53 mutants were primarily located within the hydrophobic beta-sandwich core of the DNA-binding domain, which destabilizes their folding at 37 °C. However, these mutants can regain wild-type conformation at 32 °C to 34 °C after being denatured at 37 °C. Tumor development generally selects for a loss of p53 activity; therefore, tumors in tissues maintained below 37 °C are predicted to select for fewer TS TP53 mutants. Breast cancer follows this pattern, whereas skin cancer shows an above-average frequency of TS TP53 mutations, likely due to exposure to ultraviolet radiation [14].

Studies on mouse and human p53 TS mutants demonstrated that they have potent apoptosis or cell cycle arrest activities at 32 °C, indicating that activating these mutants may produce anti-tumor effects due to their high expression levels. Since TS TP53 mutants are only expressed in tumor cells and not normal tissues, they are an attractive tumor-specific target. Hypothermia, already used as a standard of care for resuscitated cardiac arrest patients and neuroprotection in newborn infants with hypoxic-ischemic encephalopathy, has potential as a cancer treatment strategy. Lu et al. conducted an experiment to test its therapeutic potential in mice with tumor xenografts expressing TS mutant p53 [14]. Combined with chemotherapy, hypothermia demonstrated the ability to induce durable remission in a lymphoma xenograft model [14]. In addition to the hypothermia approach, recent studies showed that TS TP53 are uniquely amenable to functional rescue by structure-stabilizing drugs [13,14,15]. Despite the recent emergence of TS TP53 as a unique mutation subclass for novel rescue strategies, their prognostic significance in tumors treated with standard therapies has not been examined.

Our study stratified patients based on TP53 temperature sensitivity, as characterized by Tang et al. [13], and demonstrated a pronounced frequency of temperature-sensitive TP53 mutations within our cohort (38%), which is notably higher than the generally reported 17%. This discrepancy may stem from our exclusion of non-categorizable TP53 mutations based on the criteria set by Tang et al. [13]. The survival analysis did not reveal significant differences in overall survival between TS and temperature-insensitive TP53 mutations across the cohort when considering the 50th percentile cutoff. However, a median survival of 33 months for TI and a reduced 28 months for TS mutants were observed in the overall cohort, suggesting a potential, though not statistically significant, trend towards poorer outcomes associated with TS mutations.

Our dataset was majority male, which is in line with published esophageal cancer literature [20]. Reasons for this sexual dimorphism may include a greater incidence of high-risk behaviors in males including smoking [21] and drinking alcohol [22] and high testosterone levels [21]. Notably, when categorizing the data by gender and temperature-sensitive mutation, a stark divergence emerged; females with TS mutations exhibited a significantly reduced median survival time of 21.4 months compared to their TI counterparts at 48.0 months. This contrast was not seen in the male cohort, where TS and TI mutations correlated with equivalent median survival times of 33 months.

**Figure 2:**
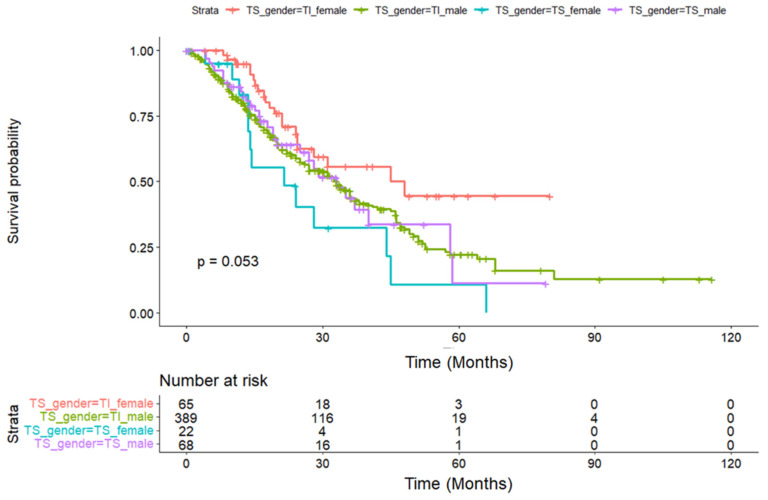
Kaplan–Meier survival curves demonstrating the impact of gender and TP53 mutation temperature sensitivity on overall survival in the adenocarcinoma patient group, at an activity of ≥50%. The curves compare the survival probabilities over time between four distinct cohorts: females with temperature-sensitive TP53 mutations (F_TS), females with temperature-insensitive TP53 mutations (F_TI), males with temperature-sensitive TP53 mutations (M_TS), and males with temperature-insensitive TP53 mutations (M_TI). *p*-values were calculated using the Kaplan–Meier estimation.

Our investigation spanned three distinct patient groups: all adenocarcinoma (‘Adeno’ group), esophageal-only adenocarcinoma (‘EC’ group), and gastric-only adenocarcinoma (‘GC’ group). This approach allowed us to discern patterns that may not have been apparent when considering gastroesophageal adenocarcinoma as a single entity. Sex-specific differences were further accentuated when examining adenocarcinoma subgroups, with a significantly greater proportion of females displaying TS mutations than males. This is substantiated by Kaplan–Meier survival analyses for four stratified groups (female TS, female TI, male TS, male TI), which demonstrated the survival disparities, particularly between females with TS and TI TP53 mutations (Figure 2). The analysis indicates a near-significant difference in survival probabilities, particularly between females with temperature-sensitive and temperature-insensitive mutations (*p* = 0.053), suggesting a potential interaction between gender and TP53 mutation temperature sensitivity that may influence patient outcomes in adenocarcinoma.

Interestingly, the use of a high stringency cutoff (at 33%) for temperature sensitivity did not produce observable differences, potentially due to the diminished number of events in the temperature-sensitive group, which underscores the need for cautious interpretation of these findings. The cohort’s characteristics—such as the predominance of males, the late-stage diagnosis in approximately 75% of patients, and the majority representation of the white/Caucasian race—align with existing literature, enhancing the credibility of our data. However, the study was limited by the preponderance of males in the cohort, which may have reduced the statistical power to detect differences between genders. Nevertheless, this observation raises the possibility of gender-specific differences in the molecular mechanisms underlying the development and progression of EC and GC. Mutation of TP53 is, in general, associated with advanced pathology and poor prognosis [23]. Since TS TP53 mutants are considered mild mutations that often have residual levels of transcriptional activity at or near 37 °C, one would have predicted that TS mutations, when examined as a subclass, may be associated with better prognosis than non-TS mutants. Our EC and GC data analysis suggests a correlation contrary to such a prediction. Research of expanded datasets and additional tumor types will be needed to verify whether TS TP53 represents a phenotypically unique class in tumor prognosis.

Although the reason for our finding is currently unclear and deserves further validation and investigation, we propose two potential rationales for this observation. Firstly, although wild-type TP53 is associated with favorable prognosis in most analyses, in certain tumors, wild-type TP53-mediated cell cycle arrest, DNA repair, or senescence is associated with an unfavorable response to chemotherapy due to interference with drug mechanisms [24,25]. Residual wild-type TP53 activity from the TS mutants may interact with treatments specific for EC and GC and produce this unfavorable outcome. Second, the TS TP53 mutant may have more robust gain-of-function oncogenic activities than non-TS mutants that promote tumor development. Since all gain-of-function studies of mutant TP53 reported to date focused on a few hotspot mutants [26,27], the gain-of-function potential of TS TP53 mutants has yet to be investigated in detail. Since gain-of-function is mediated by mass binding of mutant p53 protein to other transcription factors and altering their activity, we speculate that high levels of TS mutant p53 protein with relatively intact conformation may result in stronger gain-of-function interactions than non-TS mutants. This results in more vigorous tumor promotion and unfavorable survival.

One of the study’s strengths lies in its exploration of temperature-sensitive TP53 mutations within the EC and GC context, a novel approach that has not been previously undertaken. The study further bolsters its scope by encompassing diverse cancer types (GC and EC) and stratifying patients based on multiple factors (TP53 temperature sensitivity, history of Barrett’s esophagus, stage of cancer, sex, and race). Nevertheless, the study’s retrospective nature and potential bias from the skewed gender distribution within the cohort serve as notable limitations. The reliance on cBioportal data introduces potential biases and quality limitations regarding data accuracy and representativeness. Furthermore, the study’s inability to identify a statistically significant difference in overall survival may be attributed to the relatively modest sample size or unaccounted-for confounding variables in the analysis.

## 5. Conclusions

Taken together, the findings of this study highlight the potential importance of TS TP53 mutations in the development and progression of EC and GC. Further research is needed to validate these findings and to elucidate the underlying molecular mechanisms. Additionally, investigating the potential gender-specific differences in the prevalence and implications of TS TP53 mutations may provide valuable insights for developing more personalized treatment approaches for patients with EC and GC.

## Figures and Tables

**Table 1:** Select characteristics of the study population.

Characteristic		*n* (%)
Age at Diagnosis, mean (SD)		63.38 (11.60)
Race, *n* (freq)	White/Caucasian	483 (0.87)
	Black/African American	38 (0.069)
	Hispanic/Latino	11 (0.020)
	Asian/Other	22 (0.040)
Sex, *n* (freq)	Male	1486 (0.778)
	Female	424 (0.222)
TP 53 mutation, *n* (freq)	Temperature Sensitive	731 (0.38)
	Temperature Insensitive	1193 (0.62)
Cancer Type, *n* (freq)	Adenocarcinoma of the Gastroesophageal Junction	153 (0.08)
	Diffuse Type Adenocarcinoma of the Stomach	27 (0.014)
	Esophageal Adenocarcinoma	626 (0.33)
	Esophageal Poorly Differentiated Carcinoma	10 (0.005)
	Esophageal Squamous Cell Carcinoma	380 (0.198)
	Esophagogastric Adenocarcinoma	116 (0.06)
	Esophagogastric Cancer	175 (0.091)
	Intestinal Type Stomach Adenocarcinoma	47 (0.024)
	Mucinous Stomach Adenocarcinoma	12 (0.006)
	Papillary Stomach Adenocarcinoma	4 (0.002)
	Signet Ring Cell Carcinoma of the Stomach	8 (0.004)
	Stomach Adenocarcinoma	314 (0.163)
	Tubular Stomach Adenocarcinoma	52 (0.027)
Cancer Stage, *n* (freq)	I	22 (0.047)
	II	89 (0.190)
	III	116 (0.247)
	IV	242 (0.516)
Barrett’s Esophagus, *n* (freq)	Yes	93 (0.650)
	No	50 (0.350)

SD, standard deviation; *n*, sample size; freq, frequency.

## Data Availability

The data presented in this study are openly available in cBioportal.

## Supplementary Materials

The following supporting information can be downloaded at: https://www.mdpi.com/article/10.3390/medicina60111901/s1, Table S1: Median survival times (months) with lower and upper confidence intervals; Table S2A. Distribution of TS and TI TP53 in Barrett’s esophagus. Table S2B. Distribution of TS and TI TP53 according to cancer stage. Table S2C. Distribution of TS and TI TP53 by gender and race.

## List of Abbreviations

TP53	Tumor Suppressor Gene
TS	Temperature-Sensitive
EC	Esophageal Cancers
GC	Gastric Cancers
BE	Barrett’s Esophagus
CoxPH	Cox Proportional Hazards
CI	Confidence Interval
EAC	Esophageal Adenocarcinoma
HGD	High-Dysplasia
LGD	Low-Grade Dysplasia
IHC	Immunohistochemistry
DBD	DNA Binding Domain
UCI	Upper Confidence Interval
LCI	Lower Confidence Interval
SD	Standard Deviation
freq	Frequency
Adeno	Adenocarcinoma
NHW	Non-Hispanic White
AA	African American
H/L	Hispanic/Latino
TI	Temperature Insensitive
TS	Temperature Sensitive
F_TS	Females with Temperature-Sensitive TP53 mutations
F_TI	Females with Temperature-Insensitive TP53 mutations
M_TS	Males with Temperature-Sensitive TP53 mutations
M_TI	Males with Temperature-Insensitive TP53 mutations
GOTIT	Gastrointestinal Oncology Transformative Initiatives for Translation
IRB	Institutional Review Board
