# Supplementary material for: Differences in Gender and Overall Survival for Temperature-Sensitive TP53 Mutations in Gastroesophageal Cancer
Source: Medicina (Kaunas). 2024 Nov 20;60(11):1901. doi: 10.3390/medicina60111901 (PMC11597060; doi:10.3390/medicina60111901)
Supplement: Supplementary file 1 [file medicina-60-01901-s001.zip › medicina-3238000-supplementary.pdf]

Supplemental Table S1. Median survival times with lower and upper confidence intervals.

	n	Events	Median	0.95LCI	0.95UCI
**Adeno Group**					
TSp53 at 50% (TI)	454	215	33	28	38
TSp53 at 50% (TS)	90	43	28	24	44
TSp53 at 33% (TI)	530	252	33	28	37
TSp53 at 33% (TS)	14	6	33	20	NA
Female (TI)	65	21	48	24.3	NA
Male (TI)	389	194	33	27	37
Female (TS)	22	13	21.4	13.6	NA
Male (TS)	68	30	33	25	NA
**EC group**					
TSp53 at 50% (TI)	217	122	32.5	27	38
TSp53 at 50% (TS)	39	22	33	25	58
**GC group**					
TSp53 at 50% (TI)	112	35	NA	25.2	NA
TSp53 at 50% (TS)	26	9	NA	16.1	NA

Supplemental Table S2A. Barrett's esophagus.

TSp53 at 50%	Barrett's Esophagus	n	Freq	p-value
TI	No	31	0.316326531	0.209
TI	Yes	57	0.581632653	
TS	No	1	0.010204082	
TS	Yes	9	0.091836735	

Supplemental Table S2B. Cancer stage.

Cancer Stage	TI	Freq	TS	Freq	p-value
**Adeno Group**					
Stage 1	7	0.038888889	0	0	0.4863
Stage 2	16	0.088888889	3	0.096774194	
Stage 3	36	0.2	6	0.193548387	
Stage 4	121	0.672222222	22	0.709677419	
**EC group**					
Stage 1	5	0.084745763	0	0	0.299
Stage 2	6	0.101694915	2	0.133333333	
Stage 3	13	0.220338983	2	0.133333333	
Stage 4	35	0.593220339	11	0.733333333	
**GC group**					
Stage 1	1	0.013157895	0	0	0.7423
Stage 2	1	0.013157895	0	0	
Stage 3	13	0.171052632	2	0.181818182	
Stage 4	61	0.802631579	9	0.818181818	

Supplemental Table S2C. Gender and race.

TSp53 at 50%	Gender/Race	n	Freq	p-value
**Adeno Group**				
TI	Male	582	0.691211401	0.01245
TI	Female	124	0.147268409	
TS	Male	99	0.117577197	
TS	Female	37	0.043942993	
TI	NHW	237	0.745283019	0.1479
TI	AA	20	0.062893082	
TI	H/L	4	0.012578616	
TI	Other	9	0.028301887	
TS	NHW	45	0.141509434	
TS	AA	0	0	
TS	H/L	0	0	
TS	Other	3	0.009433962	
**EC group**				
TI	Male	306	0.759305211	0.02598
TI	Female	33	0.081885856	
TS	Male	51	0.126550868	
TS	Female	13	0.032258065	
TI	NHW	93	0.830357143	0.8334
TI	AA	1	0.008928571	
TI	H/L	1	0.008928571	
TS	NHW	17	0.151785714	
TS	AA	0	0	
TS	H/L	0	0	
**GC group**				
TI	Male	152	0.582375479	0.1404
TI	Female	64	0.245210728	
TS	Male	26	0.099616858	
TS	Female	19	0.072796935	
TI	NHW	106	0.706666667	0.2531
TI	AA	14	0.093333333	
TI	H/L	1	0.006666667	
TI	Other	8	0.053333333	
TS	NHW	18	0.12	
TS	AA	0	0	
TS	H/L	0	0	
TS	Other	3	0.02	
